# Comparable Performance of Deep Learning–Based to Manual-Based Tumor Segmentation in KRAS/NRAS/BRAF Mutation Prediction With MR-Based Radiomics in Rectal Cancer

**DOI:** 10.3389/fonc.2021.696706

**Published:** 2021-07-29

**Authors:** Guangwen Zhang, Lei Chen, Aie Liu, Xianpan Pan, Jun Shu, Ye Han, Yi Huan, Jinsong Zhang

**Affiliations:** ^1^Department of Radiology, Xijing Hospital, Fourth Military Medical University, Xi’an, China; ^2^Department of Research and Development, Shanghai United Imaging Intelligence Co., Ltd., Shanghai, China

**Keywords:** rectal cancer, deep learning, radiomics, magnetic resonance imaging, gene mutation

## Abstract

Radiomic features extracted from segmented tumor regions have shown great power in gene mutation prediction, while deep learning–based (DL-based) segmentation helps to address the inherent limitations of manual segmentation. We therefore investigated whether deep learning–based segmentation is feasible in predicting KRAS/NRAS/BRAF mutations of rectal cancer using MR-based radiomics. In this study, we proposed DL-based segmentation models with 3D V-net architecture. One hundred and eight patients’ images (T2WI and DWI) were collected for training, and another 94 patients’ images were collected for validation. We evaluated the DL-based segmentation manner and compared it with the manual-based segmentation manner through comparing the gene prediction performance of six radiomics-based models on the test set. The performance of the DL-based segmentation was evaluated by Dice coefficients, which are 0.878 ± 0.214 and 0.955 ± 0.055 for T2WI and DWI, respectively. The performance of the radiomics-based model in gene prediction based on DL-segmented VOI was evaluated by AUCs (0.714 for T2WI, 0.816 for DWI, and 0.887 for T2WI+DWI), which were comparable to that of corresponding manual-based VOI (0.637 for T2WI, *P*=0.188; 0.872 for DWI, *P*=0.181; and 0.906 for T2WI+DWI, *P*=0.676). The results showed that 3D V-Net architecture could conduct reliable rectal cancer segmentation on T2WI and DWI images. All-relevant radiomics-based models presented similar performances in KRAS/NRAS/BRAF prediction between the two segmentation manners.

## Introduction

It is clear that (1) Epidermal Growth Factor Receptor (EGFR) inhibitors could provide a beneficial clinical outcome for metastatic Colorectal Cancer (mCRC) patients with wild-type rat sarcoma viral oncogene homolog (RAS) genes rather than mutant types. However, some patients with wild-type RAS still exhibit no response to anti-EGFR therapies. To address this confusion, the downstream factors of the RAS pathway was explored, and a specific mutation in the BRAF gene (V600E) (2) was confirmed to be responsible for less response from EGFR inhibitors and a worse prognosis. Therefore, the National Comprehensive Cancer Network (NCCN) guideline (3) recommends that the genotype of KRAS/NRAS/BRAF should be determined in patients with mCRC and further claims that patients with these mutations should not be provided with medication such as cetuximab or panitumumab, either alone or in combination with other anticancer drugs, since there is little chance of them having any benefit and the toxicity and expense suffered will not be reasonable.

Up to now, it is still a state-of-the-art routine practice to detect gene mutation status by pathologically analyzing biopsy samples or resected tissues. However, there is a growing recognition (4) that tissue-based genetic tests have some limitations such as intratumoral heterogeneity, clonal evolution, and poor DNA quality, especially in biopsy samples, which can lead to a suboptimal profile of tumor genetic characteristics and be of limited value in routine practice. In recent years, liquid biopsy has emerged to be an alternative method to determine gene status. However, this newly raised technology is still limited for clinical practice due to the availability of samples for testing, the non-standardized method, and the low sensitivity in low-stage tumor (5). Therefore, efficient identification of RAS and BRAF status in rectal cancers using a non-invasive method, which could feasibly reveal the whole tumor gene features in real-time, would be of meaningful assistance in providing individual tailored therapy.

There have been a certain number of researches based on PET/CT (6), CT (7), or MRI (8) focusing on detecting RAS gene mutations in rectal cancer, while these studies all delineated tumors manually. It is worth noting that the inherent limitations of manual segmentation, such as long time-consumption and inter- and intra-observer variability, have significant impact on medical image quantitative analysis (9) and the efficacy and safety of the radiotherapy plan (10). Fortunately, state-of-the-art auto segmentation based on deep-learning architecture has been developed and shown to be able to address these problems. Successful application included making differential diagnosis in brain (11) and contouring gross tumor volumes in rectal cancer radiotherapy (12). For 3D medical image segmentation, 3D V-Net, a special fully convolutional neural network (CNN), has been shown to be able to produce satisfactory segmentation results (13). The network first detects the boundary from a “coarse” resolution, then provides accurate spatial localization through a “fine” resolution.

Radiomics, with its high-throughput quantitative image features, has shown exciting power in assessing treatment response (14), genetic profile (8), predicting lymph node (15), and distant metastasis (16) in respect to rectal cancers. Furthermore, combinations of DL-based automatic segmentation and radiomics have been demonstrated with great potential in glioma grading (17), treatment response assessment (18), and the isocitrate dehydrogenase-1 (IDH1) mutation prediction (19) of glioblastoma. However, the combination of DL-based auto segmentation with MR-based radiomics in predicting gene mutation for rectal cancer has not been investigated. Thus, we attempt to segment rectal cancer *via* 3D V-Net on T2WI and DWI and then compare the performance of radiomics in predicting the KRAS/NRAS/BRAF status between DL-based auto segmentation and manual-based segmentation.

## Materials and Methods

### Dataset

**Figure 1:**
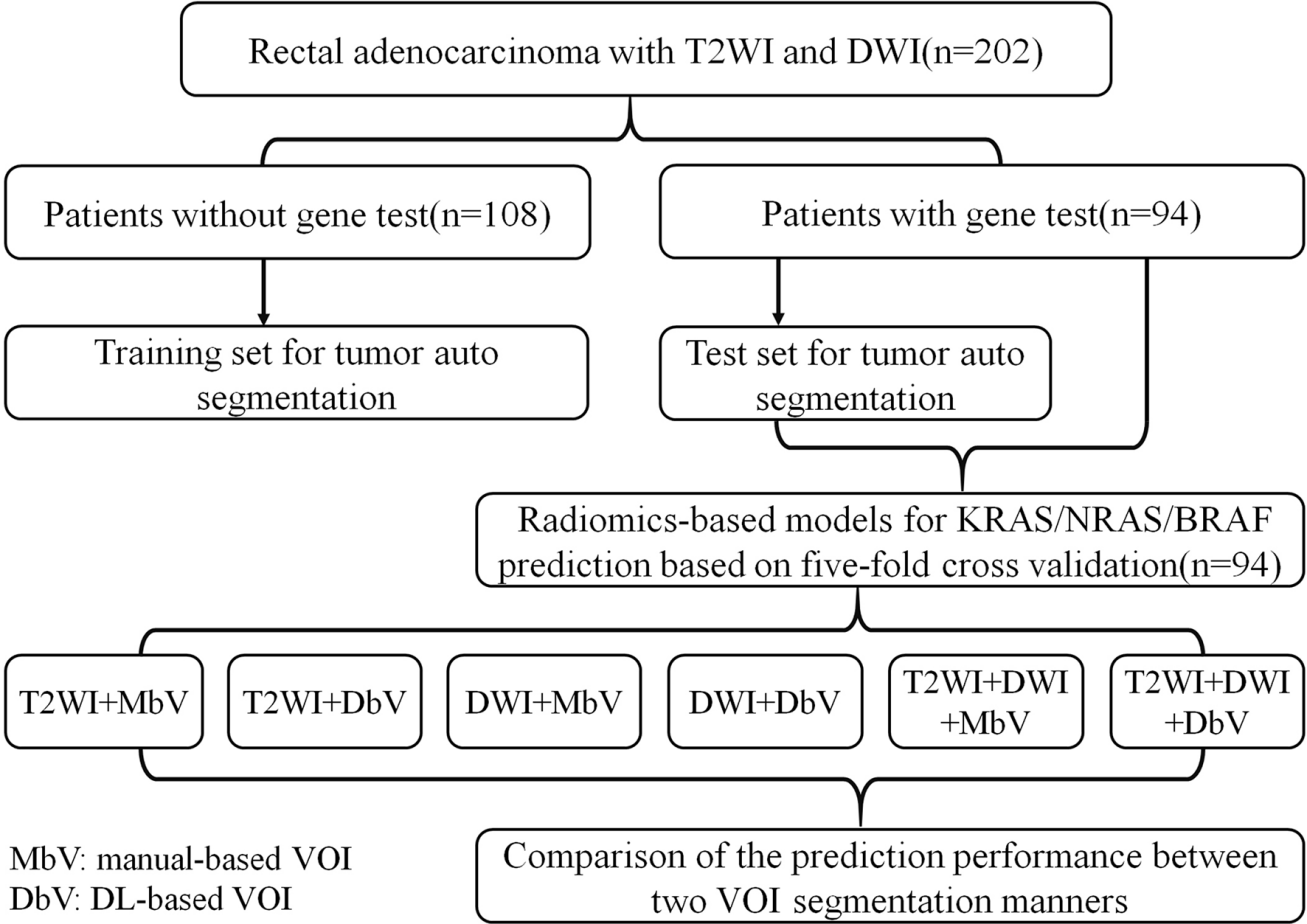
Experiment flow chart. VOI, volumes of interest.

This retrospective study was approved by the institutional review board in our hospital, and informed patient consent was waived. A total of 202 participants (mean age 59.88 ± 11.82 years, 139 males and 63 females) with rectal adenocarcinoma confirmed by colonoscopy biopsy were recruited from 333 patients who had underwent pelvic MR imaging on a 3.0T scanner (November 2016 to May 2019) after screening according to the following exclusive criteria: (a) treated with any strategy before MR imaging or surgery (n=75); (b) the interval between MR imaging and postoperative pathology was more than 4 weeks (n=8); (c) gross artifacts or severe distortion of MR images (n=18); (d) absence of visible lesion or the volume of lesion was less than 1 cm^3^ on MR image (n=7); (e) other pathological types of tumor (mucinous adenocarcinoma, neuroendocrine carcinoma, and malignant melanoma) (n=23). Among the 202 participants, 94 patients were subject to a KRAS/NRAS/BRAF mutation test, and the interval was less than 4 weeks between MR imaging and the gene test. Among the 94 patients who underwent the gene test, 53 patients harbored mutant KRAS/NRAS/BRAF, and 41 patients were wild type. The remaining 108 patients were not tested for mutations and could not be used to assess mutation prediction, but they were suitable for modeling segmentation. Therefore, we used the 108 patients without the gene test as the training set for the auto segmentation model and the 94 patients with the gene test as the test dataset, each including both the T2WI and DWI images. The radiomics-based model for gene mutation prediction was constructed based on 94 patients’ MR images *via* 5-fold cross validation. Considering the different imaging modalities and tumor segmentation manners, we constructed six radiomics-based models, which were T2WI+manual-based VOI, T2WI+DL-based VOI, DWI+manual-based VOI, DWI+DL-based VOI, T2WI+DWI+manual-based VOI, and T2WI+DWI+DL-based VOI. The detailed experiment flow chart is shown in **Figure 1**, and patients’ baseline clinical characteristics for genotype prediction is summarized in **Table 1**.

**Table 1:** Patient baseline characteristics for genotype (KRAS/NRAS/BRAF) prediction.

Characteristics	Wild type (n = 41)	Mutant type (n = 53)	*P*
Age, years (Mean ± SD)	60.44 ± 12.93	61.57 ± 10.30	0.639
Gender, n (%)			0.297
Male	29 (70.7%)	32 (60.4%)	
Female	12 (29.3%)	21 (39.6%)	
Histologic grade, n (%)			0.206
Well	5 (12.2%)	9 (17.0%)	
Moderate	35 (85.4%)	38 (71.7%)	
Poor	1 (2.4%)	6 (11.3%)	
pT stage, n (%)			**0.021**
T1/2	22 (53.7%)	16 (30.2%)	
T3/4	19 (46.3%)	37 (69.8%)	
pN stage, n (%)			0.183
N0	25 (61.0%)	25 (47.2%)	
N1	16 (39.0%)	28 (52.8%)	
CEA, n (%)			0.543
≤5 ng/ml (normal)	27 (65.9%)	38 (71.7%)	
>5 ng/ml (abnormal)	14 (34.1%)	15 (28.3%)	
CA-199, n (%)			0.588
≤27 u/ml (normal)	35 (85.4%)	43 (81.1%)	
>27 u/ml (abnormal)	6 (14.6%)	10 (18.9%)	

Chi-squared or Fisher’s exact tests, as appropriate, were used to compare the differences in categorical variables, while independent samples t test was used to compare the differences in age. Bold value: Rectal cancer with more advanced T stage is prone to evolve mutant KRAS/NRAS/BRAF (P=0.021). p, pathological.

### MR Image Acquisition

All MR scanning was performed on a 3.0T MR scanner (Discovery MR750, GE Medical Systems) with an eight-channel phased-array coil. Bowel preparation was implemented by drinking folium sennae soup (a kind of laxative) after dinner the night before the examination. Antispasmodic and other intestinal contrast agents were not used. Rectal MRI protocols included axial T1WI (TR/TE = 487/8 ms), coronal and sagittal T2WI (TR/TE = 7,355/136 ms), oblique axial small FOV FRFSE T2WI (TR/TE = 6,055/130 ms, Slice Thickness = 3 mm, Gap = 0.3 mm, FOV=200 × 200 mm, Matrix = 352×256), and axial single-shot EPI DWI (TR/TE = 4,734/80 ms, Slice Thickness = 4 mm, Spacing = 0.5 mm, FOV=340 × 340 mm, Matrix = 128×140, NEX = 8, b = 0, 1,000 s/mm^2^). An oblique axial T2WI high-resolution sequence was planned perpendicularly to the bowel with the tumor, while the axial DWI sequence was performed parallelly to the horizontal line.

### Imaging Pre-Processing

As the reliability of manual VOI delineation had been reported in our previous study (20), the whole-tumor volume was manually delineated as the ground truth annotation on T2WI and DWI (b=1,000 s/mm^2^) images by one radiologist with 8 years of experience in abdominal MRI and scrutinized by another senior abdominal MRI radiologist with 20 years of experience. The regions of contiguous normal rectal wall and lumen against tumor were manually labeled on T2WI images, and the magnetic susceptibility artifacts were labeled on DWI images, which were used for the training and validation of the automated tumor segmentation algorithm. All manual delineations were performed using ITK-SNAP (version 3.8) (21). Because of the peristalsis of rectum and different imaging parameters such as matrix, FOV (Field of View), slice thickness, and scan position line, the processing of the registration and image fusion between T2 and DWI images was not performed.

All MR images were normalized to accelerate the convergence of neural network training. First, the MR images were resampled to the same spatial resolution: 0.4×0.4×3.3 (mm), and then the gray values were linearly normalized into the range [0, 1]. Considering the GPU memory, the input 3D patch size was set to 96×96×32. Due to the limited amount of training images, image augmentation was performed, which included shift, rotation, scale, and flip slightly.

### Network Architecture of 3D V-Net

**Figure 2:**
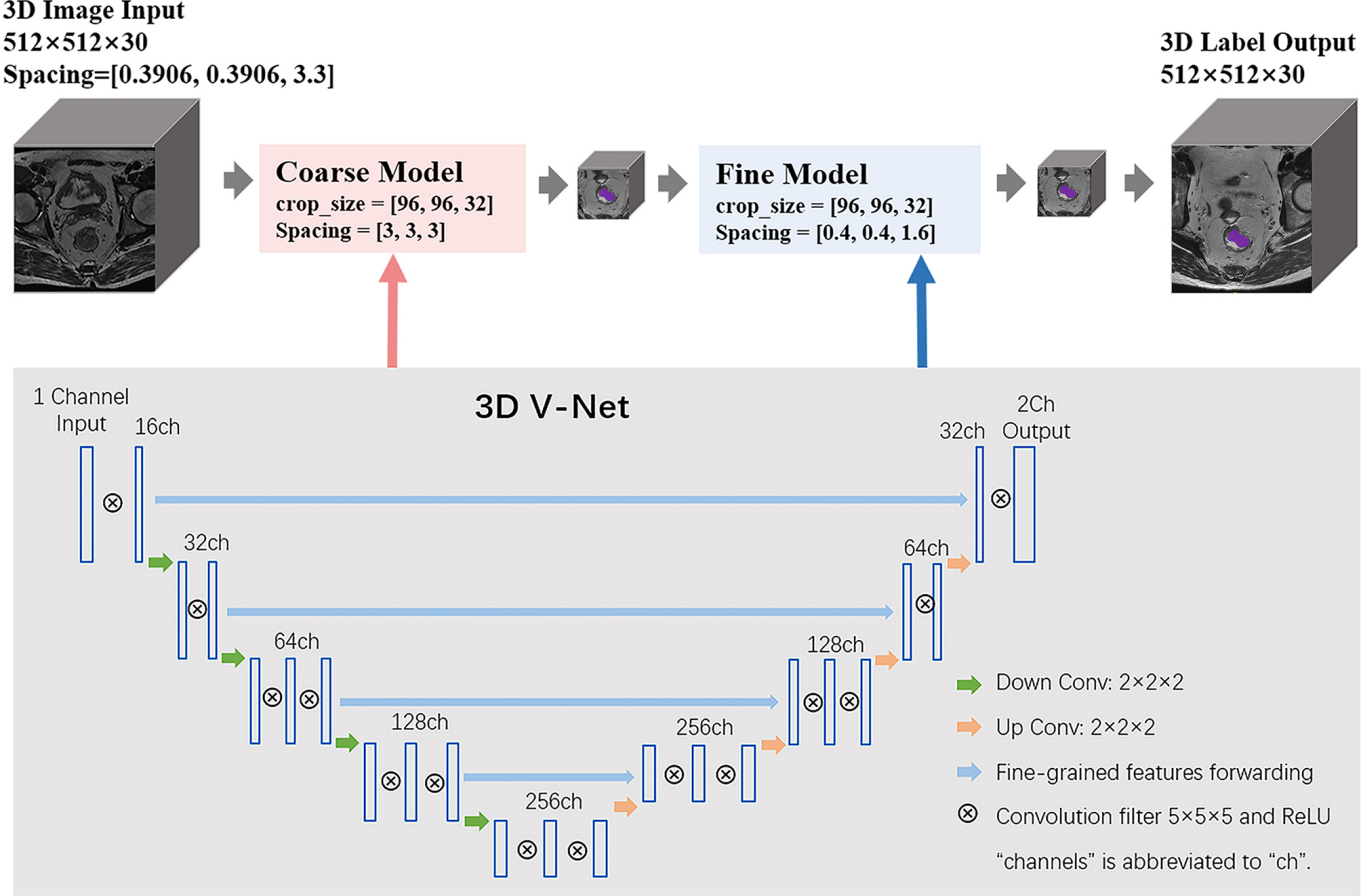
The schematic network architecture of cascade V-Net.

We applied cascade learning in this work based on 3D V-Net for the tumoral tissue segmentation of the rectum on T2WI and DWI sequences. The code of 3D V-Net was improved from the V-Net (13). The architecture of the conventional V-Net has two pathways: the left part of the network consists of a compression path, while the right part decompresses the signal until its original size is reached. The detailed network architecture is shown in **Figure 2**. The proposed cascade neural network includes one coarse model and one fine model. The coarse-to-fine segmentation method detects the boundary from coarse resolution to the highest fine resolution to provide accurate spatial localization. The input of the 3D V-Net is a single sequence of a patient such as T2WI, while the output is a map of classification probability, which determines whether voxels of image belong to tumor or background. The loss function based on the Dice coefficient (range [0, 1]), which we sought to maximize, was performed in the training process. It is defined as

$$\text{D}=\frac{2\sum_{i}^{N}p_{i}g_{i}}{\sum_{i}^{N}p_{i}^{2}+\sum_{i}^{N}g_{i}^{2}}$$

Where *N* is the number of voxels of the image, *p_i_* is the prediction probability of the *i*-th voxel which belongs to the target region, and *g_i_* denotes whether the *i*-th voxel belongs to ground truth annotation or not (1 means yes, 0 means no). The volume size of the input and output image is 512×512×30, and the parameters of spacing for the coarse model and fine model are [3,3,3] and [0.4,0.4,1.6], respectively. Similar to other CNN, the training process was iterated with min-batch and stochastic gradient descent to ensure quick convergence. Tumor volume was segmented using forward propagation in the test process.

### Genes (KRAS/NRAS/BRAF) Mutational Status Analysis

The tissue blocks were acquired from resected tumors, and pathologists selected the samples for gene mutational analysis. Genomic DNA was extracted from 5 mm formalin-fixed, paraffin-embedded (FFPE) tumor tissue sections, using a DNA FFPE Tissue Kit (AmoyDx, China). KRAS (exons 2, 3, and 4), NRAS (exons 2, 3), and BRAF (exons 15, V600E) mutations were detected by using polymerase chain reaction (PCR) and amplification-refractory mutation system (ARMS). Among the 53 patients with mutant genes, 48 patients were KRAS mutation, four patients were NRAS mutation, and one was BRAF mutation.

### Radiomics Features Extraction, Selection, and Classifier Modeling for Gene Mutation Prediction

Radiomics analysis was performed by a clinical research platform (uAI Research Portal, United Imaging Intelligence Co., Ltd, China). The code for radiomics analysis was developed based on pyradiomics (https://pyradiomics.readthedocs.io/en). First, a total of 2,600 features were extracted from the labeled tumor volume of each MR sequence. These features were computed by the combination of 104 original image features with 25 image filters. The original image features include First-order, Shape, Gray Level Co-occurrence Matrix (GLCM), Gray Level Run Length Matrix (GLRLM), Gray Level Size Zone Matrix (GLSZM), Gray Level Dependence Matrix (GLDM), and Neighborhood Gray-Tone Difference Matrix (NGTDM). The image filters consist of Gaussian noise, curvature flow, Laplacian of Gaussian, Discrete Gaussian, Speckle noise, Recursive Gaussian, shot noise, and Wavelets. Second, feature selection was performed on the extracted features (2,600 dimensions) by least absolute shrinkage and selection operator (Lasso) method to work out an optimal feature subset (around 10 dimensions, for example). We set two parameters for LASSO, the feature scaler and shrinkage penalty, as min-max scaler and 0.02, respectively. The selected features for each radiomics-based model are presented in the supplementary material. Then, a radiomics-based model was built by support vector machine (SVM) classifier with the selected features. The parameters of SVM consist of penalty factor C (3.0), Gamma (0.03), and kernel (radial basis function). The predict models were verified by five-fold cross-validation and thus derived an average performance.

### Statistics

Differences of patient baseline characteristics between the wild-type and mutant groups were tested using independent samples *t* test and chi-squared or Fisher’s exact tests, as appropriate. Performance of the V-Net with respect to tumor segmentation was evaluated in the test dataset using the Dice coefficient. The AUC (area under the curve), accuracy, sensitivity, and specificity were calculated to evaluate the performance of the radiomics-based model in differentiating gene status. DeLong’s test was used to compare two AUCs of the manual based model and deep learning–based model of identical imaging modality. The statistical analyses were conducted with SPSS (version 26.0), Medcalc (version 20.0), and PyCharm (version 2018, Python version 3.0). A two-sided *p* value < 0.05 was statistically considered significant difference.

## Results

### Performance of 3D V-Net Segmentation Algorithm

**Figure 3:**
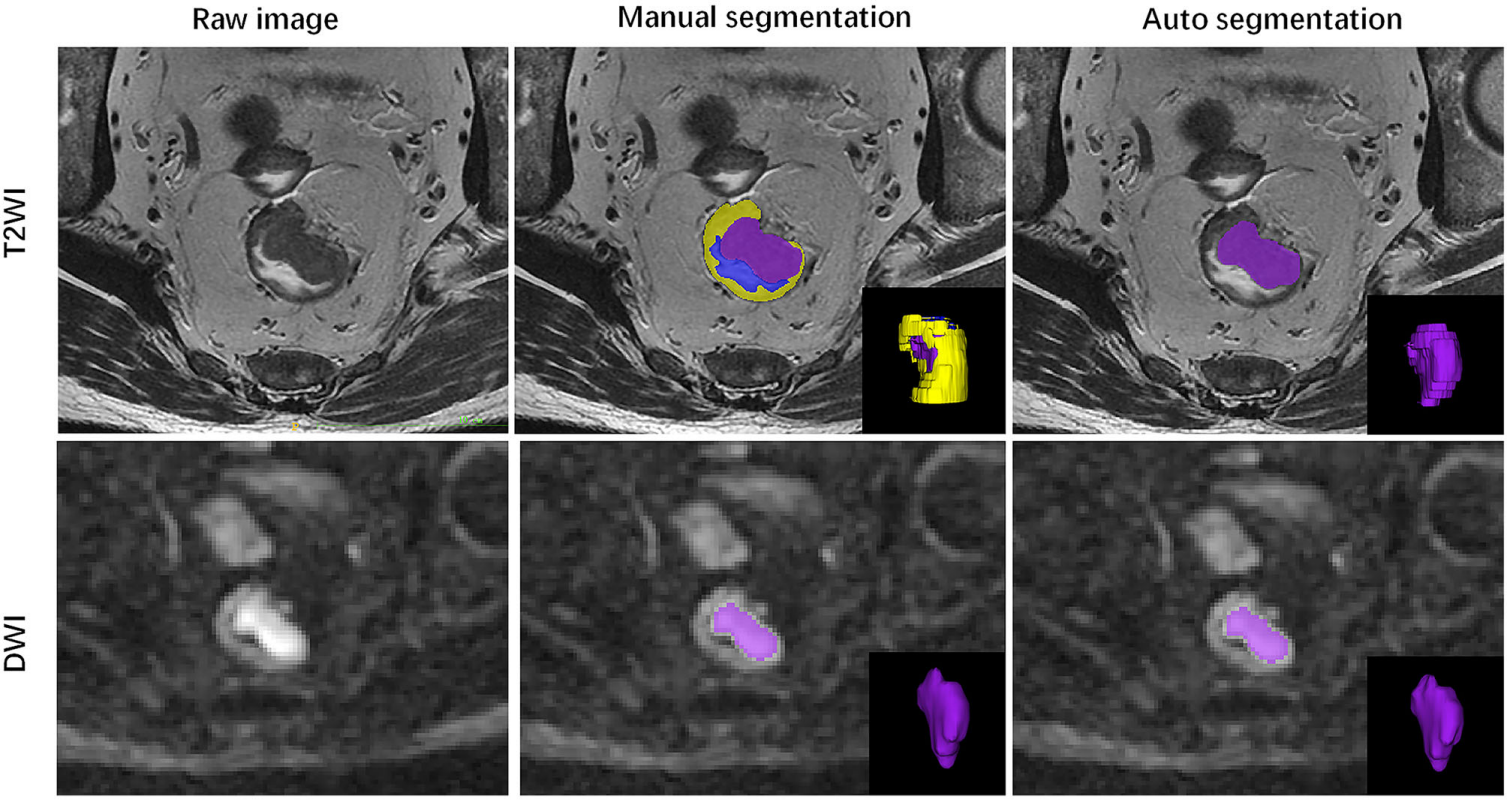
Illustration of automated segmentation using 3D V-Net *versus* ground truth on rectal MR images of a 51-year-old male. Purple indicates tumor, yellow indicates normal rectal wall, and blue indicates lumen. The Dice was 0.980 on T2WI and 0.981 on DWI for this patient.

The ground truth annotation includes 202 rectal cancers on T2WI and DWI sequences. To evaluate the performance of the 3D V-Net, the Dice Similarity Coefficient (DSC) was used to compare segmentations between AI and a radiologist. The volumetric segmentations generated from the deep learning model are probability maps. The mean and standard deviation of the Dice is 0.878 ± 0.214 and 0.955 ± 0.055 for T2WI and DWI separately in the test dataset. A paradigm of tumor segmentation results are shown in **Figure 3**.

### Clinical and Pathological Characteristics

Among the 202 participants, 94 patients underwent a KRAS/NRAS/BRAF mutation test. There were 53 patients who harbored mutant genes, and 41patients were wild type. A statistical difference in terms of age, gender, histologic grade, pN stage, CEA, and CA-199 levels was not found between wild-type and mutant groups, except at the pT stage (*p* = 0.021). It seems that a tumor with more advanced T stage is prone to evolve mutant gene (**Table 1**).

### Testing of Gene Mutation Prediction With Radiomics Signature

**Figure 4:**
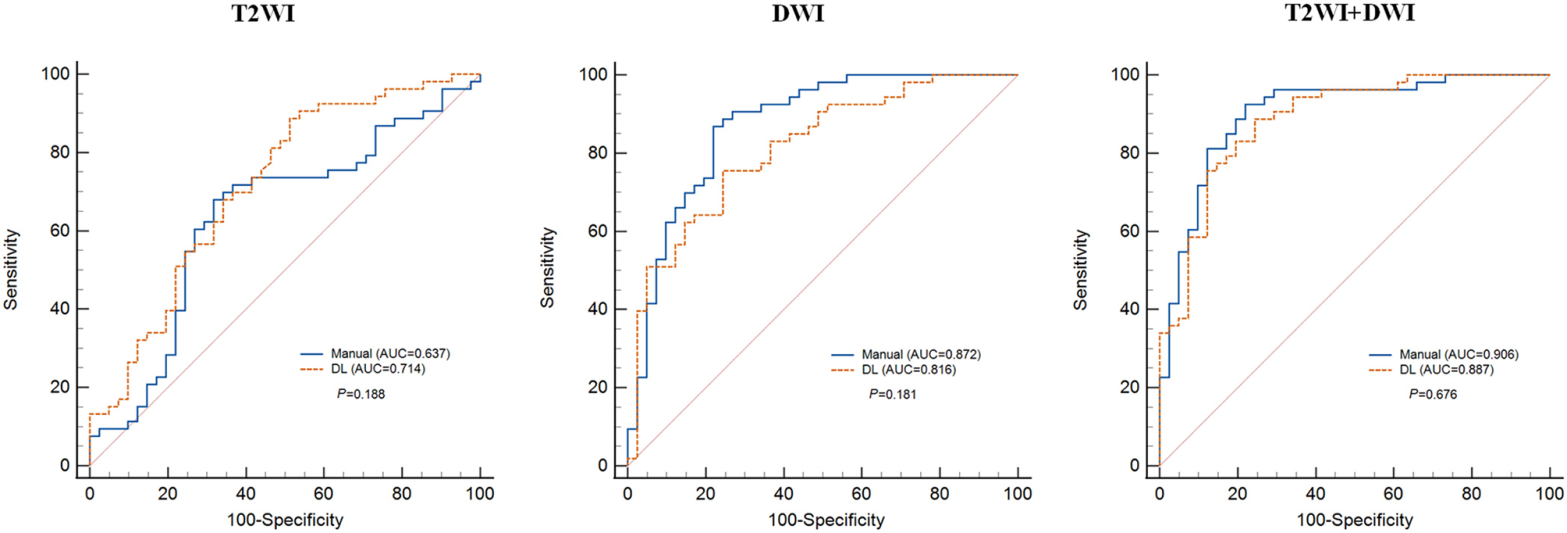
Mean receiver operating characteristics (ROC) curve of five-fold cross validation for each radiomics-based model. DL, deep learning; AUC, area under the curve.

We built radiomics-based models with extracted features from two MR sequences of T2WI and DWI. Each sequence was processed separately to compute features from DL-based and manual-based VOI, respectively. Furthermore, we combined all features computed from T2WI and DWI sequences, and then applied the feature selection method LASSO to obtain an optimal feature subset. Thus, in total we collected six feature subsets and built six radiomics-based models for gene prediction. The mean performance of each model based on five-fold cross-validation is listed in **Table 2** and includes accuracy, specificity, sensitivity, and AUC. For each imaging modality, the prediction performance of gene mutation did not show any statistical difference between DL-based segmentation and manual-based segmentation (**Table 2** and **Figure 4**).

**Table 2:** Performance of the radiomics-based models in predicting genotype (KRAS/NRAS/BRAF).

Imaging modality	VOI	Accuracy	Specificity	Sensitivity	AUC	*P*
T2WI	Manual	0.669	0.614	0.716	0.637	0.188
	DL	0.674	0.464	0.744	0.714	
DWI	Manual	0.776	0.731	0.809	0.872	0.181
	DL	0.711	0.678	0.736	0.816	
T2WI+DWI	Manual	0.829	0.803	0.847	0.906	0.676
	DL	0.783	0.661	0.882	0.887	

For each model, the mean performance from five-fold cross-validation is presented in this table. DeLong’s test was used to compare the two AUCs of the manual-based model and the deep learning–based model for identical imaging modality. DL, deep learning; VOI, volumes of interest; AUC, area under the curve.

## Discussion

In this study, we segmented rectal cancer *via* 3D V-Net on T2WI and DWI and then compared the radiomics performance in predicting KRAS/NRAS/BRAF status between DL-based auto segmentation and manual-based segmentation. By virtue of volumetric convolution and coarse-to-fine segmentation models, higher tumor segmentation performance (Disc=0.878 and 0.955 for T2WI and DWI) was achieved by V-Net in our study compared with Trebeschi’s (22) (Dice=0.70 for confusion image of T2WI and DWI) and Wang’s (Dice=0.74 for T2WI) (12) work. This could be explained with low signal noisy ratio caused by 1.5T MR scanner in Trebeschi’s work, volumetric information loss with 2D U-net architecture in Wang’s work, and their relatively small sample size (n=140 and 93, respectively). It has been widely recognized that qualified standard input image data are crucial for training CNN architecture to obtain high performance (23). We recruited MR images from 202 rectal patients who underwent 3.0T MR scans, which ensured eligible input data with high signal noise ratio and spatial resolution. Furthermore, we manually labeled regions of contiguous normal rectal wall and lumen against tumor on T2WI images and the magnetic susceptibility artifacts on DWI image. This process is distinctive to previous work (12, 22) and helpful to confirm the boundary of VOI.

Though the T2WI had higher spatial resolution, the higher Dice was achieved on DWI. The noise, intensity non-uniformity, partial volume averaging, and tumor background contrast were key elements to influence the accuracy of segmentation (24). Compared to T2WI, the tumor background contrast and intensity uniformity on DWI were greater, which may facilitate the computer to identify and recognize the tumor region. As there was greater non-uniformity of intensity and low tumor background contrast on T2WI, especially in respect to muscle, bladder, and normal rectal wall, which may present similar signal intensity and texture to tumorous tissue, we found that after intensity histogram match there were still two samples that totally failed to give the correct segmentation (Disc=0). One of them put the segmentation label on the right piriformis, and the other put the segmentation label on the uterus (**Supplementary Figure 1**). Except for intensity non-uniformity and low tumor background contrast on T2WI, limited training samples may be another reason that contributes to failed segmentations.

The ultimate goal of auto segmentation is to facilitate clinical or experimental application. Since the genotype (KRAS/NRAS/BRAF) is strongly correlated with response to anti-EGFR therapies (25), we evaluated the reliability and usefulness of auto segmentation with radiomics analysis on these genotype predictions. No matter whether referring to single imaging modality or combined imaging modality, we found that the performance of genotype prediction is similar between manual-based and DL-based segmentation (**Table 2**). For example, the AUC is 0.906 for manual-based and 0.887 for DL-based VOI in combination of T2WI and DWI features on the test dataset (*P*=0.676). When referring to radiomics analysis, the genotype prediction performance of DWI is superior to that of T2WI, and combination modality surpasses any single imaging modality no matter whether it is manual-based VOI or DL-based VOI (**Table 2**). The KRAS/NRAS/BRAF are the downstream effectors of the EGFR signal pathway involved in tumor cell proliferation, differentiation, and invasion (26). Tumors with mutant genes more likely exhibit greater aggressiveness and angiogenesis, which will result in faster progress, worse survival, and lower apparent diffusion coefficient (ADC) value (27). The DWI can indicate the functional information of tissue by evaluation of water molecular mobility, which is estimated with ADC value, while T2WI are prone to indicate anatomic information, which might explain the higher genotype prediction performance of DWI compared to that of T2WI. Cui and his colleagues (8) developed a radiomics signature to predict KRAS mutations with moderate performance on T2WI (AUC=0.682 for internal validation and 0.714 for external validation), which is concordant to our genotype prediction performance with T2WI (AUC=0.714, DL-based VOI). We noted that for T2WI modality, the AUC of the radiomics-based model with DL-based VOI is higher than that of manual-based VOI (0.714 vs 0.637). In theory, the manual segmentation is the ground truth for radiomics analysis. So, the performance of the DL-based model should not be superior to manual-based VOI. To assess the difference of gene prediction performance between these two models, a Delong’s test was used, and the result showed no statistical significance (*P*=0.181). We speculate that limited sample size may be one reason. On the other hand, DL-based VOI may contain some peritumoral region, which could exhibit an inflammatory response and tumor microinvasion. The inflammatory response and tumor microinvasion may provide additional information that is related to gene mutation. Meng et al. (28) investigated a radiomics-based model in predicting the KRAS-2 genotype based on multiparametric MRI (T1WI, T2WI, DWI, and DCE) with 0.651 of AUC in the validation cohort, which is slightly inferior to our combination model (T2WI+DWI, AUC=0.878 for manual VOI) and may be attributed to low signal noise ratio and spatial resolution of their 1.5T MR scanner. Several studies have demonstrated the value of CT radiomics (7) (AUC = 0.829) or texture analysis (AUC = 0.82) (29) or PET/CT (AUC = 0.684 ~ 0.75) (30) on genotype prediction of KRAS/NRAS/BRAF or KRAS alone. Compared with CT or PET/CT, MRI can be of benefit with no concern about radiation exposure and contrast agent injection and simultaneously provide a wonderful detailed tissue contrast.

Though 202 patients were involved in our analysis, it is still necessary to validate this framework and compare it with different architectures, such as the recently developed Generally Nuanced Deep Learning Framework (31), in larger and diverse datasets. Currently, all segmentation acquired with deep learning architecture should be carefully reviewed before being submitted for further application, especially for making a radiotherapy plan. The requirement of high-quality annotated data is a great challenge for auto segmentation, which needs a standard imaging protocol, strict quality control, and accurate annotation. For rectum DWI, magnetic susceptibility artifact is the main obstacle that affects the accuracy of auto segmentation. Therefore, we labeled the artifact on DWI of the training dataset. If possible, labeling all anatomic structures and artifacts on the training dataset will definitely improve the performance of deep learning architecture, but that will be a huge workload. Considering the great performance of combined imaging modality on predicting genotype, further investigation of combining CT and MRI is needed.

## Conclusions

In this study, 3D V-Net architecture provided reliable rectal cancer segmentation on T2WI and DWI compared with expert-based segmentation, and auto segmentation was subjected to radiomics analysis in the prediction of KRAS/NRAS/BRAF mutation status and may produce a good prediction result.

## Data Availability Statement

The original contributions presented in the study are included in the article/**Supplementary Material**. Further inquiries can be directed to the corresponding authors.

## Ethics Statement

The studies involving human participants were reviewed and approved by institutional review board of Xijing hospital. Written informed consent for participation was not required for this study in accordance with the national legislation and the institutional requirements.

## Author Contributions

GZ and LC produced the manuscript. GZ and LC conceived and designed framework of this article. GZ, LC, AL, XP, JS, and YeH collected and analysed the data. JZ and YiH supervised this study and reviewed the manuscript. All authors contributed to the article and approved the submitted version.

## Funding

This research was funded by Key Research and Development Projects in Shaanxi, grant number 2018ZDXM-SF-059.

## Conflict of Interest

Authors LC, AL and XP were employed by Shanghai United Imaging Intelligence Co., Ltd.

The remaining authors declare that the research was conducted in the absence of any commercial or financial relationships that could be construed as a potential conflict of interest.

## Publisher’s Note

All claims expressed in this article are solely those of the authors and do not necessarily represent those of their affiliated organizations, or those of the publisher, the editors and the reviewers. Any product that may be evaluated in this article, or claim that may be made by its manufacturer, is not guaranteed or endorsed by the publisher.
