# Supplementary material for: Comparable Performance of Deep Learning–Based to Manual-Based Tumor Segmentation in KRAS/NRAS/BRAF Mutation Prediction With MR-Based Radiomics in Rectal Cancer
Source: Front Oncol. 2021 Jul 29;11:696706. doi: 10.3389/fonc.2021.696706 (PMC8358773; doi:10.3389/fonc.2021.696706)
Supplement: Supplementary file 1 [file DataSheet_1.docx]

Supplementary Material

# Supplementary Data

Selected features with Lasso for each radiomics-based model.

**Model 1（T2WI+manual-based segmentation）：**

| Feature | Coefficient |
| --- | --- |
| wavelet_glcm_wavelet-hlh-idmn | 0.124401525 |
| wavelet_glcm_wavelet-hhl-maximumprobability | 0.10870678 |
| log_glcm_log-sigma-2-mm-3d-imc2 | -0.039076917 |
| mean_gldm_largedependencelowgraylevelemphasis | -0.04426307 |
| log_glcm_log-sigma-1-5-mm-3d-imc2 | -0.06598039 |
| wavelet_glcm_wavelet-lhh-idn | -0.108295925 |
| specklenoise_glcm_idn | -0.114273593 |
| log_firstorder_log-sigma-0-5-mm-3d-maximum | -0.124326326 |

**Model 2（T2WI+DL-based segmentation）：**

| Feature | Coefficient |
| --- | --- |
| wavelet_glcm_wavelet-hhl-maximumprobability | 0.040855188 |
| mean_gldm_largedependencelowgraylevelemphasis | -0.05419265 |
| wavelet_ngtdm_wavelet-hlh-contrast | -0.05467365 |
| log_glcm_log-sigma-1-0-mm-3d-autocorrelation | -0.05550676 |
| wavelet_gldm_wavelet-lhl-smalldependencehighgraylevelemphasis | -0.05722538 |
| specklenoise_firstorder_kurtosis | -0.0602198 |
| log_glcm_log-sigma-1-5-mm-3d-imc2 | -0.078768395 |
| log_glcm_log-sigma-2-mm-3d-imc2 | -0.1085088 |

**Model 3（DWI+ manual-based segmentation）：**

| Feature | Coefficient |
| --- | --- |
| wavelet_glcm_wavelet-hhh-contrast | 0.18197085 |
| wavelet_glcm_wavelet-hlh-imc2 | 0.1628888 |
| wavelet_glszm_wavelet-hhl-sizezonenonuniformitynormalized | 0.063920714 |
| specklenoise_glcm_correlation | 0.008594499 |
| recursivegaussian_glszm_sizezonenonuniformitynormalized | -0.002571523 |
| wavelet_glszm_wavelet-hhh-lowgraylevelzoneemphasis | -0.00734679 |
| log_gldm_log-sigma-0-5-mm-3d-largedependencelowgraylevelemphasis | -0.011647437 |
| wavelet_glcm_wavelet-llh-inversevariance | -0.07589187 |
| log_glszm_log-sigma-1-0-mm-3d-sizezonenonuniformitynormalized | -0.1745713 |

**Model 4（DWI+ DL-based segmentation）：**

| Feature | Coefficient |
| --- | --- |
| wavelet_glcm_wavelet-hhh-contrast | 0.07102735 |
| normalize_glszm_smallareaemphasis | 0.037317257 |
| wavelet_glcm_wavelet-hlh-imc2 | 0.023788607 |
| wavelet_firstorder_wavelet-llh-90percentile | 0.023297686 |
| wavelet_gldm_wavelet-hhl-lowgraylevelemphasis | 0.00897516 |
| normalize_glszm_smallareahighgraylevelemphasis | 9.36E-17 |
| normalize_glszm_smallarealowgraylevelemphasis | 9.36E-17 |
| log_glszm_log-sigma-2-mm-3d-smallareaemphasis | -0.012600386 |
| log_glszm_log-sigma-1-0-mm-3d-smallarealowgraylevelemphasis | -0.07857406 |
| boxsigmaimage_gldm_largedependencelowgraylevelemphasis | -0.09014684 |
| log_gldm_log-sigma-0-5-mm-3d-largedependencelowgraylevelemphasis | -0.120577373 |
| log_glszm_log-sigma-1-0-mm-3d-sizezonenonuniformitynormalized | -0.1234248 |
| boxsigmaimage_glszm_zonevariance | -0.15208514 |

**Model 5（T2WI+DWI+ manual-based segmentation）：**

| Feature | Coefficient |
| --- | --- |
| wavelet_glcm_wavelet-hlh-imc2_dwi | 0.266430855 |
| wavelet_glcm_wavelet-hhh-contrast_dwi | 0.1881637 |
| wavelet_glszm_wavelet-hhl-sizezonenonuniformitynormalized_dwi | 0.08556522 |
| wavelet_glcm_wavelet-hlh-idmn_t2 | 0.083964534 |
| log_gldm_log-sigma-0-5-mm-3d-largedependencelowgraylevelemphasis_dwi | -0.004295259 |
| shotnoise_glszm_zonevariance_t2 | -0.005292323 |
| wavelet_glszm_wavelet-hhl-sizezonenonuniformity_t2 | -0.0099923 |
| recursivegaussian_glszm_sizezonenonuniformitynormalized_dwi | -0.012958328 |
| wavelet_gldm_wavelet-lhl-smalldependencehighgraylevelemphasis_t2 | -0.026103174 |
| log_glszm_log-sigma-1-0-mm-3d-sizezonenonuniformitynormalized_dwi | -0.131499857 |
| log_glcm_log-sigma-2-mm-3d-imc2_t2 | -0.272591025 |

**Model 6（T2WI+DWI+ DL-based segmentation）**

| Feature | Coefficient |
| --- | --- |
| wavelet_glcm_wavelet-hlh-imc2_dwi | 0.08891208 |
| wavelet_glcm_wavelet-hhh-contrast_dwi | 0.028106626 |
| boxsigmaimage_glcm_correlation_dwi | -0.010721243 |
| log_glszm_log-sigma-1-0-mm-3d-sizezonenonuniformitynormalized_dwi | -0.04011746 |
| boxsigmaimage_gldm_largedependencelowgraylevelemphasis_dwi | -0.054566875 |
| wavelet_gldm_wavelet-lhl-smalldependencehighgraylevelemphasis_t2 | -0.054806884 |
| log_glszm_log-sigma-1-0-mm-3d-smallarealowgraylevelemphasis_dwi | -0.06311303 |
| boxmean_glszm_largeareahighgraylevelemphasis_dwi | -0.07259564 |
| log_gldm_log-sigma-0-5-mm-3d-largedependencelowgraylevelemphasis_dwi | -0.1314508 |
| boxsigmaimage_glszm_zonevariance_dwi | -0.1483834 |
| wavelet_ngtdm_wavelet-hlh-contrast_t2 | -0.2339616 |
| log_glcm_log-sigma-2-mm-3d-imc2_t2 | -0.281128317 |

## Supplementary Figure


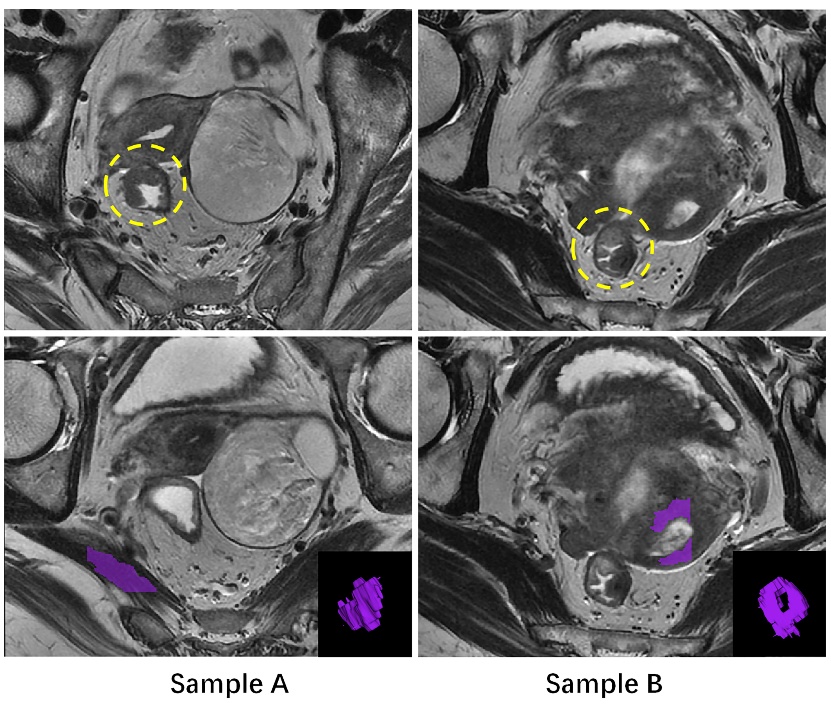


**Supplementary Figure 1.** DL-based auto segmentation model put the label on the right piriformis of sample A and put the label on uterus of sample B. Yellow dashed circle indicates the rectal cancer, purple region indicates the wrong labeled VOI with DL-based auto segmentation.
